# Evaluation of in vitro biocompatibility of human pulp stem cells with allogeneic, alloplastic, and xenogeneic grafts under the influence of extracellular vesicles

**DOI:** 10.1038/s41598-023-39410-0

**Published:** 2023-08-01

**Authors:** Marius Heitzer, Qun Zhao, Johannes Greven, Philipp Winnand, Xing Zhang, Felix Marius Bläsius, Eva Miriam Buhl, Michael Wolf, Sabine Neuss, Frank Hildebrand, Frank Hölzle, Ali Modabber

**Affiliations:** 1 Department of Oral and Maxillofacial Surgery, University Hospital of RWTH Aachen, Pauwelsstraße 30, 52074 Aachen, Germany; 2 Department of Orthopedics, Trauma and Reconstructive Surgery, University Hospital of RWTH Aachen, Pauwelsstraße 30, 52074 Aachen, NRW Germany; 3 Institute of Pathology, University Hospital of RWTH Aachen, Pauwelsstraße 30, 52074 Aachen, NRW Germany; 4 Department of Orthodontics, University Hospital of RWTH Aachen, Pauwelsstraße 30, 52074 Aachen, NRW Germany; 5 BioInterface Group, Helmholtz Institute for Biomedical Engineering, RWTH Aachen University, Pauwelsstraße 20, 52074 Aachen, NRW Germany

**Keywords:** Medical research, Stem-cell research

## Abstract

Therapies using dental pulp stem cells (DPSCs) or stem cell-derived extracellular vesicles (EVs) have shown promising applications for bone tissue engineering. This in vitro experiment evaluated the joint osteogenic capability of DPSCs and EVs on alloplastic (maxresorp), allogeneic (maxgraft), and xenogeneic (cerabone) bone grafts. We hypothesize that osteogenic differentiation and the proliferation of human DPSCs vary between bone grafts and are favorable under the influence of EVs. DPSCs were obtained from human wisdom teeth, and EVs derived from DPSCs were isolated from cell culture medium. DPSCs were seeded on alloplastic, allogeneic, and xenogeneic bone graft substitutes for control, and the same scaffolds were administered with EVs in further groups. The cellular uptake of EVs into DPSC cells was assessed by confocal laser scanning microscopy. Cell vitality staining and calcein acetoxymethyl ester staining were used to evaluate cell attachment and proliferation. Cell morphology was determined using scanning electron microscopy, and osteogenic differentiation was explored by alkaline phosphatase and Alizarin red staining. Within the limitations of an in vitro study without pathologies, the results suggest that especially the use of xenogeneic bone graft substitutes with DPSCS and EVs may represent a promising treatment approach for alveolar bone defects.

## Introduction

Bone defects of the alveolar ridge usually occur as a result of tooth loss, inflammation, trauma, or pathology and pose major problems during dental or implant rehabilitation. Augmentation procedures thus aim to generate sufficient bone volume for implant placement^1^. Different bone grafting materials can be used for the augmentation of bone defects. Because of its osteogenetic, osteoinductive, and osteoconductive properties, bone grafts of autologous origin represent the gold standard for the therapy of bony defects in the human jaw, although they are associated with major drawbacks such as donor-side morbidity^2^, multiple required surgeries, and increased operation times^3^. In addition to autologous bone, allogeneic, xenogeneic, and alloplastic bone graft substitutes (BGSs) can be used for the augmentation of bone defects. Bone substitutes offer the advantage of eliminating harvest morbidity, but they are currently inferior to autologous bone in terms of osteogenesis, osteoinduction, and osteoconduction. Much research has thus been conducted to improve the properties of BGSs.

Multipotent mesenchymal stem cells (MSCs) are adult stem cells that can be isolated from a wide variety of tissues^4–7^. In 2000, MSCs were isolated for the first time from the healthy pulp tissue of extracted third molars^8^. In addition to their rapid proliferation and colony-forming abilities^9^, MSCs derived from DPSCs have indicated strong differentiation potential to osteoblasts, adipocytes, chondrocytes, odontoblasts, and neurons^10^. Compared to MSCs derived from bone marrow, DPSCs have a higher proliferation rate^10^, are clonogenic cells, and have promising regenerative potential to treat severe tissue damage, all of which make DPSCs a frequently studied stem cell type among MSCs in research. The ability to differentiate into bone-forming cells make DPSCs a promising therapeutic option in the regenerative medicine of bone deficiency^3,11^.

Recent evidence suggests that the beneficial regenerative effects of stem cell-based therapy are most likely orchestrated by their paracrine processes^9,12–17^. EVs are secreted by cells in a paracrine manner, are surrounded by a lipid bilayer structure, and have a diameter of about 150–180 nm. They contain genetic substances such as microRNAs (miRNAs) and EVs are part of cell-to-cell communication in the form of exosomes or microvesicles^18–21^. MicroRNAs are non-coding RNA components that inhibit the translation of mRNA. Thus, they are an elementary component of cell proliferation and cell differentiation^22^. The target specificity and modulatory genes of miRNAS are related to osteogenesis, and consequently promote osteogenic differentiation of MSCs and thus induce bone tissue regeneration^23^.

Impairment by degradative enzymes has often limited the use of miRNAs in bone remodeling studies in the past^24^. These limitations could be overcome by the production of naturally derived EVs, since these vesicles can be used to transport miRNAs to target cells^25^. EVs have the advantage of being easily obtained from body or cell culture fluids^26,27^, and they are characterized by stability with low susceptibility, elicit little immune response if any, and have an intrinsic homing effect, which favors the uptake of EVs with osteogenic miRNAs by target cells^28^. New approaches to stem cell therapy are thus being expanded to include the use of EVs^9,13^.

Considering that EVs and DPSCs play a major role in cell-based therapies^16^, and that EVs can potentially modulate the proliferation and differentiation of DPSCs in osteogenesis, an investigation of DPSCs on different scaffolds and EV interactions seems essential to the development of new therapies for bone defects. Currently, an insufficient number of studies have investigated the osteogenic cell response of DPSC under the influence of EVs on different conventional BGSs in vitro. The aim of this study was thus to investigate the internalization of EVs into DPSCs and to determine the influence on the attachment, growth, and osteogenic differentiation of DPSCs on alloplastic, xenogeneic, and allogeneic bone scaffolds under the influence of EVs.

## Material and methods

### DPSC extraction and cell culture

As previously described^29^, DPSCs were generated from 3 impacted third molars extracted in healthy adult males in the Oral and Maxillofacial Surgery Department of RWTH Aachen University. All methods were carried out in accordance with relevant guidelines and regulations of the ethical approval by the Ethics Committee of the Medical Faculty of RWTH Aachen University (EK 374/19). Written informed consent was obtained from all participants. The teeth were placed in chlorhexidine before being opened with sterile dental fissure drills at the cementoenamel junction. Sterile barbed broaches were used to dissect pulp tissue from the crown and root. Prior to the isolation of DPSC, a mixed enzymatic solution was prepared using 1 ml of collagenase type I (12 mg/ml), 1 ml of dispase (16 mg/ml) in 2 ml of sterile phosphate-buffered saline (PBS) containing 100 mg/ml of penicillin, and 100 mg/ml of streptomycin. The minced pulp tissue was transferred into the prepared enzyme solution for digestion for 60 min at 37 °C and vortexed every 30 min. Using a 70 μM cell sieve, any large-cell aggregates were removed to obtain a single-cell suspension. Digestion was terminated by the addition of 3 ml of minimal essential medium (MEM) containing 10% (v/v) fetal bovine serum (FBS). The single-cell suspensions were then centrifuged at 1,000 rpm for 5 min at room temperature. After removal of the supernatant, the pellets were resuspended in 1 ml of cell medium and cultured in a 25 cm^2^ cell culture dish at 37 °C in an incubator at 5% CO_2_ atmosphere and 100% relative humidity. After approximately 3–5 days, the adherent DPSCs migrated outward from the pulp-tissue pieces. Briefly, the isolated DPSCs were cultured in EV-free basic MEM medium (growth medium) containing 10% (v/v) FBS and 1% (v/v) antibiotic–antimycotic solution at 37 °C in an incubator with 5% CO_2_ atmosphere and 100% relative humidity. Refreshing of the medium was performed every two days. DPSCs in stages 3 to 5 were used in the subsequent trials. Induction of osteogenic differentiation was conducted by culturing DPSCs in osteogenic differentiation medium supplemented with 0.1 mM ascorbic acid, 100 nM dexamethasone, and 10 mM β-glycerophosphate.

### DPSC-derived EV isolation and examination

After reaching roughly 80% confluence, DPSCs were rinsed twice with PBS and cultivated for another 24 h in medium without FBS. To remove cellular debris, and any unattached cells, the supernatants were collected, differentially centrifuged (300 g, 2.000 g, and 5.000 g) for 15 min at 4 °C, and filtered with a 0.22 μm filter; the sediment was then discarded. The supernatant was then transferred to an ultracentrifuge tube and subjected twice to ultracentrifugation at 20,000 g for 90 min at 4 °C to pellet DPSC-derived EVs. The pellets were re-suspended in 100 μL of sterile PBS to obtain homogeneous EV suspension and then stored at − 80 °C for further application.

**Figure 1:**
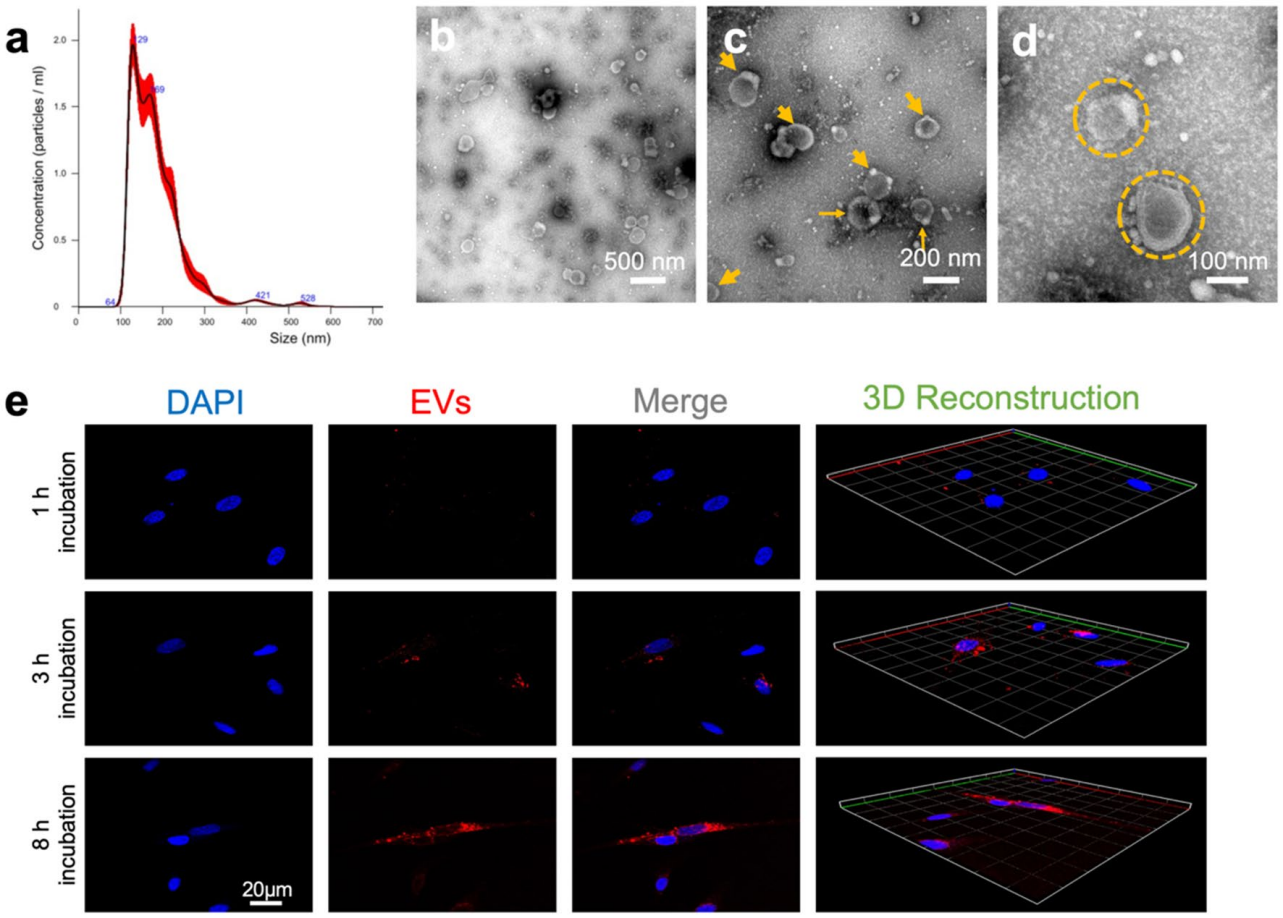
Examination of characteristics of DPSC-derived EVs: The number and sizes of EVs were analyzed by (**a**) nanoparticle tracking analysis (NTA) with NanoSight NS300. (**b**–**d**) The EVs shape were examined by transmission electron analysis (TEM). **e** Illustrates the cellular internalization of WSA-labeled EVs (red fluorescence) into DAPI positive DPSC cells (blue fluorescence) after 1 h, 3 h and 8 h.

Nanoparticle tracking analysis (NTA) was then used to test quantitative concentration and the particle size distribution of DPSC-derived EVs. All samples were diluted to a final volume of 1 mL using PBS. A pre-test was performed to evaluate the ideal measurement concentration of 20–100 particles per frame; see Fig. 1a. Settings were implemented according to the manufacturer’s software manual (NanoSight NS300). In addition, the morphology and size of the isolated DPSC-EVs were assessed by transmission electron microscopy (TEM) (Fig. 1b–d). A total of 10 μL of DPSC-EVs was pelleted, resuspended in 10 μL of HEPES buffer, and dropped onto a carbon-coated copper grid for TEM observations.

### Bone grafts

Commercially available particulate bone grafts (particle size of 0.5–2 mm) were used in this in vitro study. We used maxresorb (botiss biomaterials GmbH, Germany) as a biphasic calcium phosphate (BCP) alloplastic scaffold. For the allogeneic scaffold, we used maxgraft (botiss biomaterials GmbH, Germany), a freeze-dried bone allograft. For the xenogeneic bone graft, we used cerabone (botiss biomaterials GmbH, Germany), a deproteinized bovine bone mineral.

### Internalization of DPSC-derived EVs

First, the isolated DPSC-derived EVs were fluorescently labeled with a molecular lectin probe, Wheat Germ Agglutinin Conjugates (WGA, Alexa Fluor 488 conjugate), for 30 min at room temperature before being terminated by centrifugation at 20,000 g for 90 min and resuspended in PBS. These WGA-labeled EVs were co-cultured with DPSC (5 × 10^4^/confocal dish) for different times (1 h, 3 h, and 8 h); the cells were then rinsed with PBS and fixed in 4% paraformaldehyde. The cell nucleus was then stained with 6-diamidino-2-phenylindole (DAPI) (cat. no. 2381700, Invitrogen, USA) for another 15 min. The cellular uptake of WGA-labeled EVs by DPSCs after different incubation times was observed using confocal laser scanning microscopy (CLSM). Additionally, 3D reconstruction images from WGA and DAPI fluorescence were automatically formed using ZEN lite software at different time points (Fig. 1e).

### Cytoskeleton and morphology assessment under DPSC-EVs intervention

After culturing for 7 days, the cellular morphologies on various bone scaffolds under EV intervention at an initial density of 20,000 cells/dish were evaluated through cytoskeleton staining and scanning electron microscopy (SEM). For the assessment of cytoskeletal organization, the cell-scaffold samples were pre-treated with EVs and afterward rinsed twice with PBS and fixed in 4% paraformaldehyde for 20 min, followed by a pre-treatment with Triton X-100 (0.1% v/v) for 5 min. The samples were then co-stained with rhodamine-phalloidin (cat. no. R415, Invitrogen, Germany) to visualize F-actin and DAPI (cat. no. 2381700, Invitrogen, Germany) to visualize the nuclei for 45 min and 5 min, respectively. Sample visualization was conducted after 1 h, 3 h, and 8 h via CLSM, and Z-stack images for 3D construction were obtained.

For SEM observations, the cell-scaffold constructs in various groups were gently washed twice with PBS buffer, fixed with 2.5% glutaraldehyde for 4 h, and then dehydrated with a series of different concentrations of ethanol (30%, 50%, 70%, 90%, and 100%) twice. Next, cell/scaffold constructs were dried overnight according to an established protocol^30^ and then sputtered with Au/Pd and observed using SEM (XL30 FEG; FEI, Eindhoven, the Netherlands) with an acceleration voltage of 10 kV.

### Cell proliferation and attachment on bone grafts

Afterward, the cell-seeded BGSs were cultivated in confocal dishes at an initial density of 20,000 cells/dish for 3 or 7 days. Half the samples underwent EV stimulation (5 × 10^9^ particles/mL). At each predetermined time point, the medium was discarded and the cells were washed three times. Two different stainings were conducted, per the manufacturer’s instructions: (1) live cell calcein AM staining (cat. no. PK-CA707-30002, PromoKine, Germany) and (2) DAPI staining (cat. no. 2381700, Invitrogen, USU). DPSC cells growing on the bone scaffolds were rinsed three times and observed via CLSM to determine the proliferative effect.

### Cell viability assessment

A Live/Dead Cell Assay Kit (PromoKine, PromoCell GmbH, Germany) was used to detect cell viability from intracellular esterase activity and the plasma membrane integrity of DPSCs. DPSCs were seeded on to the three different bone graft scaffolds (20,000 cells/dish) in the osteogenic medium for 1, 3, 5, and 7 days. The scaffolds were then rinsed twice with warm PBS, and the cells were labeled with a mixed fluorescence dye containing 2 μM calcein AM and 4 μM ethidium homodimer-III (EthD-III) for 45 min, per the manufacturer’s instructions. Finally, the ratio of green fluorescence to red fluorescence was analyzed using ImageJ software. (Free Java software was provided by the National Institutes of Health, Bethesda, MD, USA.)

### Osteogenic differentiation determination

Alkaline phosphatase (ALP) staining was used to evaluate the early- and late-phase osteogenic differentiation in all groups after 7 and 14 days of cultivation. In brief, the culture medium was aspirated, and DPSCs were gently washed with PBS buffer twice. A fixing solution was added and then incubated at room temperature. After 5 min, the fixing solution was aspirated, and ALP staining solution (cat. no. GR3358974-1, Abcam Co., UK) was added in darkness and incubated at room temperature for a further 15 min. Observation of ALP-positive (ALP+) cells was conducted via optical microscopy and analyzed using ImageJ software.

Alizarin red staining (ARS) (cat. no. 3512879, Merck, Germany) was used to determine the mineralized nodule formation of late-phase osteogenic differentiation by calcium deposition. After osteogenic incubation for 14 days, the cells in various groups were rinsed twice with PBS, fixed in 4% paraformaldehyde for 10 min, and then stained with 0.1% ARS solution (40 mM, pH 4.2) in darkness for 25 min at room temperature. These mineralized precipitates were observed and photographed via an optical microscope and analyzed using ImageJ software. The mineralized nodules appeared as a dark-red center and a light-red peripheral area.

### Statistical analysis

Analyses were performed by using Prism 7.0 (GraphPad Software, Inc., San Diego, CA, USA). Before analysis, the values were tested using D’Agostino–Pearson or Shapiro–Wilk test criteria for normal distribution and passed the Brown–Forsythe test for equal variances. A two-way ANOVA was used to analyze the parametric data of the groups with respect to two different categorical variables: Variable one includes the different bone material and variable two the presence or absence of EVs. A post hoc Tukey’s multiple comparison test, with individual variances computed for each comparison, was conducted. The data in this paper represent the means ± standard deviation (SD). Differences between the groups were considered significant when *p* < 0.05.

## Results

The yield of EVs obtained from DPSCs had a measured concentration of 2 × 10^9^ nanoparticles/ml. Confirming the vesicle size and morphology of EVs obtained in the study presented here showed similarities with values in the literature, NTA via NanoSight NS300 and TEM were used to characterize the EVs. The SEM images illustrated that the EVs had a typical cup-shaped morphology with different hydrodynamic diameters (Fig. 1b–d). NTA revealed EVs in the size range of about 184.9 ± 67.0 nm. The SEM images also showed that the isolated EVs had similar shapes, which together proved that the isolation methods had resulted in homogenous EV batches.

### Internalization of DPSC-derived EVs

Figure 1e shows the interaction of the WGA-labeled EVs and the DPSCs with DAPI-stained nuclei. After an incubation period of 1 h, few EVs were initially taken up into the cytoplasm of the DPSCs. Few WSA-positive EVs were evident near the DAPI-stained nuclei. Over the consecutive duration of 3–8 h, we noted a steady uptake and an increasing accumulation of EVs into the cytoplasmic space.

### Cytoskeleton and morphology assessment under DPSC-EV intervention

**Figure 2:**
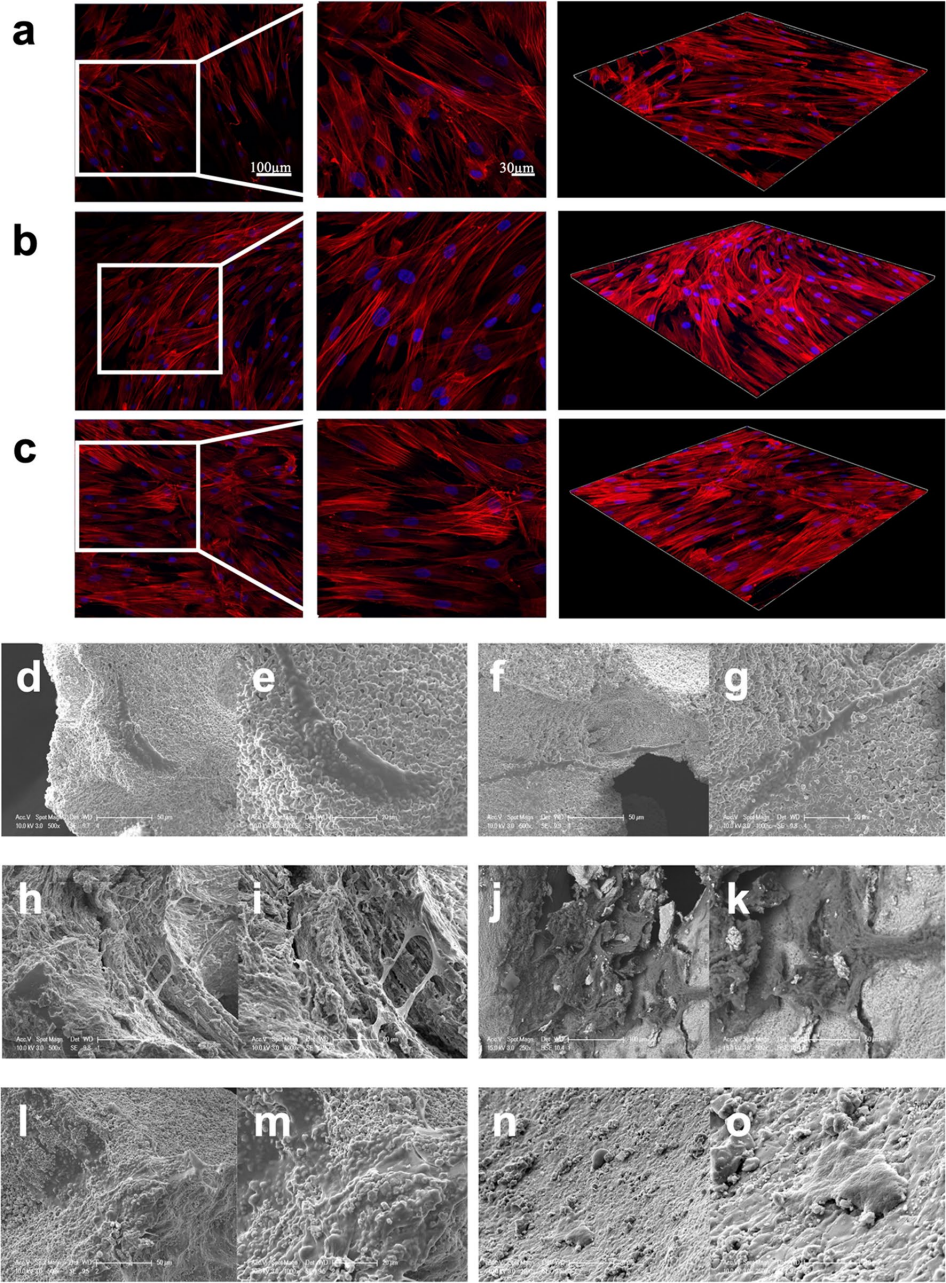
(**a**–**c**) Shows a homogeneous distribution of actin fibres and filament elongation of DPSC under the influence of EVs on (**a**) alloplastic, (**b**) allogeneic and **c** xenogeneic scaffolds. The magnifications of the left images were 400 × and the middle images 600 × . The images on the right are 3D reconstructions. (**d**–**o**) Photographs of scanning electron microscopy from day 7 of the DPSC cell cultures. (**d**, **e**) show DPSC on alloplastic scaffold. (**f**, **g**) DPSC + EVs on alloplastic scaffold. (**h**, **i**) shows DPSC on allogeneic scaffold. (**j**, **k**) DPSC + EVs on allogeneic scaffold. (**l**, **m**) show DPSC on xenogeneic scaffold. (**n**, **o**) DPSC + EVs on xenogeneic scaffold.

In monocultured DPSC with EVs, an abundance of F-actin fibers were observed in all the bone graft materials under the influence of EVs. The fluorescent images shown in Fig. 2a–c indicate a homogeneous distribution of actin fibers and filament elongation of DPSCs on alloplastic, allogeneic, and xenogeneic BGSs. Morphologically, no difference was evident between the groups in the staining of the actin of the cytoskeleton of DPSCs under the influence of EVs.

SEM images from day 7 (Fig. 2) of the DPSC cell cultures showed normal shapes of MSCs on the BGSs. The cells on the alloplastic (d–g), allogeneic (h–k), and xenogeneic (l–o) BGSs showed normal expansion and a spindle-shaped morphology, first with EVs and second in the absence of EVs. The cells were oriented along the surface of the grafts and grew in random regions.

### Cell proliferation and attachment on bone grafts

**Figure 3:**
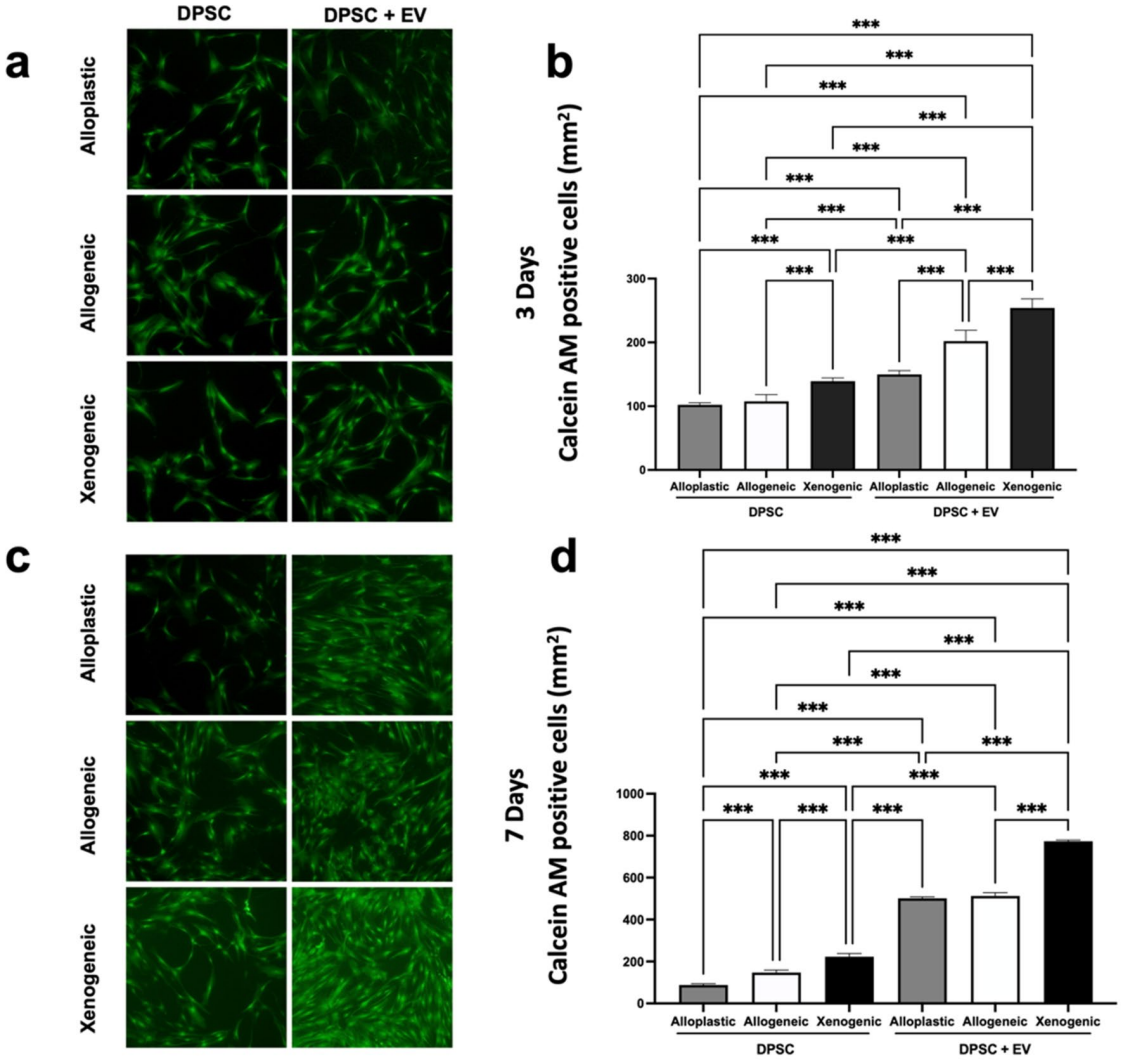
Measurement of cell proliferation and attachment by Calcein AM staining; (**a**, **b**) after 3 days of cell culture. (**c**, **d**) after 7 days of cell culture. 50 × magnification; ***p < 0.001.

DPSC viability was evaluated with and without stimulation by EV nanoparticles on different BGSs using a calcein AM assay. Table 1 and Fig. 3 show the measurement of cell proliferation and attachment by the fluorescence of calcein at days 3 and 7. The number of calcein AM–positive (henceforth CAM+) cells increased from day 3 to day 7 in all groups. In addition, for both time points, DPSCs with the presence of EVs resulted in significantly more CAM+ cells (*p* < 0.001). At approximately 773.25 ± 5.95 CAM+ cells/mm^2^, the most significantly abundant (*p* < 0.001) CAM+ cells were detected at 7 days in the DPSCs and EVs in the xenogeneic BGSs, compared to the alloplastic BGSs (with 501.38 ± 6.86 CAM+ cells/mm^2^) and allogeneic BGSs (with 512.88 ± 15.45 CAM+ cells/mm^2^).

**Table 1:** Quantitative data of cell proliferation.

Groups	Cell proliferation 3 days (CAM+ cells/mm^2^)	Cell proliferation 7 days (CAM+ cells/mm^2^)
DPSC		
Alloplastic	102.1 ± 3.18	87.75 ± 6.11
Allogeneic	107.6 ± 10.65	147.3 ± 12.28
Xenogeneic	139.1 ± 5.33	223 ± 15.34
DPSC + EVs		
Alloplastic	149.8 ± 5.99	501.4 ± 6.87
Allogeneic	202.1 ± 16.85	512.9 ± 15.45
Xenogeneic	245 ± 14.2	773.3 ± 5.946

### Cell viability assessment

**Figure 4:**
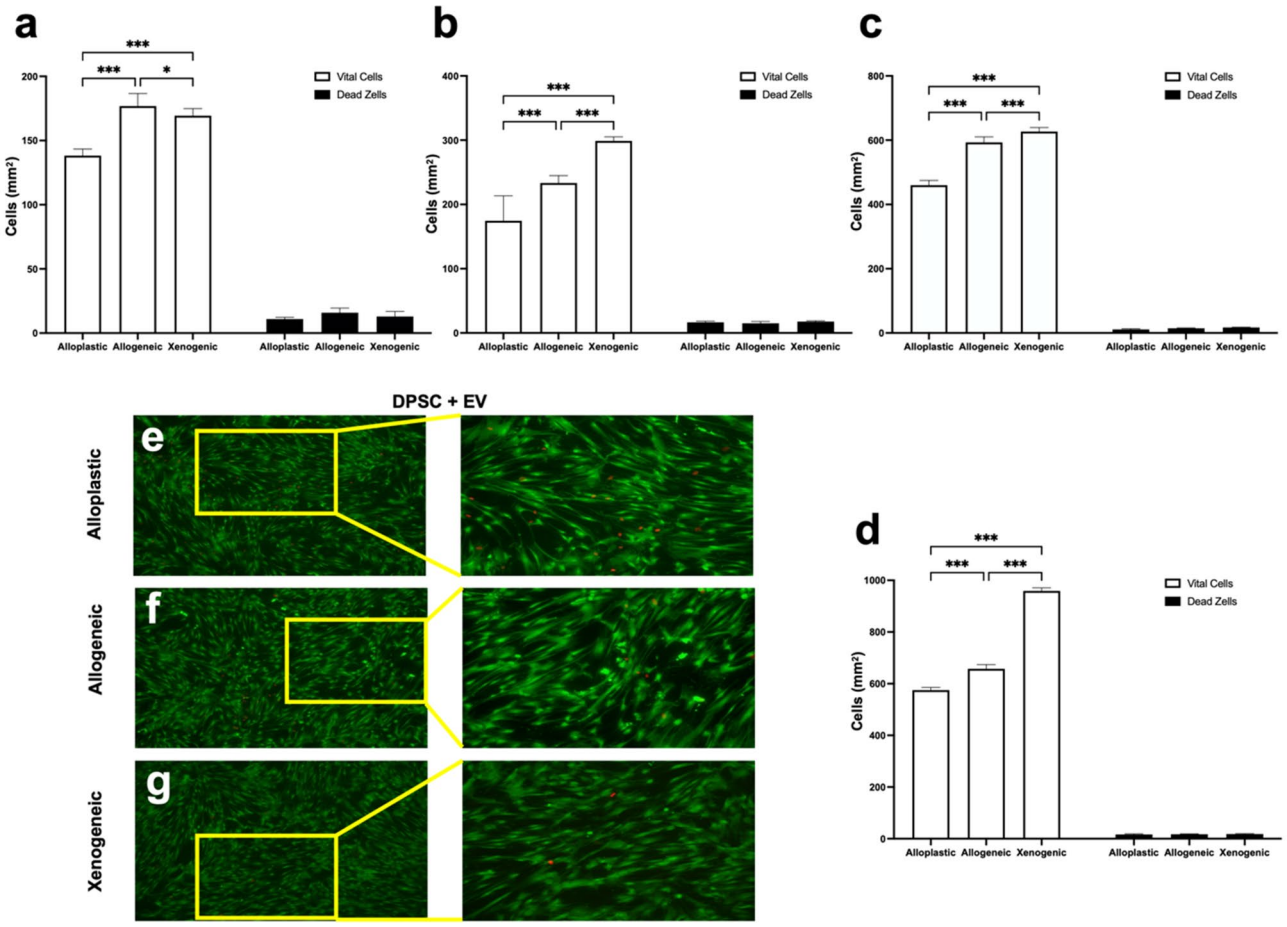
Graphical illustration of the live-and-death evaluation of DPSC + EVs after (**a**) 1, (**b**) 3, (**c**) 5, and (**d**) 7 days. ***(*p* < 0.001); *(*p* < 0.05). (**e**–**f**) Florescence images of live-dead staining of DPSC + EVs after 7 days. Left image 50 × magnification; right image 100 × magnification.

We next tested the viability of DPSC under the influence of EVs cultured for 1, 3, 5, and 7 days in osteogenic medium on alloplastic, allogeneic, and xenogeneic BGSs (Fig. 4). At all observation periods, very few dead cells were noted in the live/dead assay, which did not increase analogously with the growing cell number of live cells. For the significantly highest number of 959 ± 11.81 live cells per mm^2^, the xenogeneic group had 18.00 ± 1.69 dead cells per mm^2^ after 7 days. At all four time points, no significant difference was noted in the number of dead cells per mm^2^ between all groups.

### Osteogenic differentiation determination

**Figure 5:**
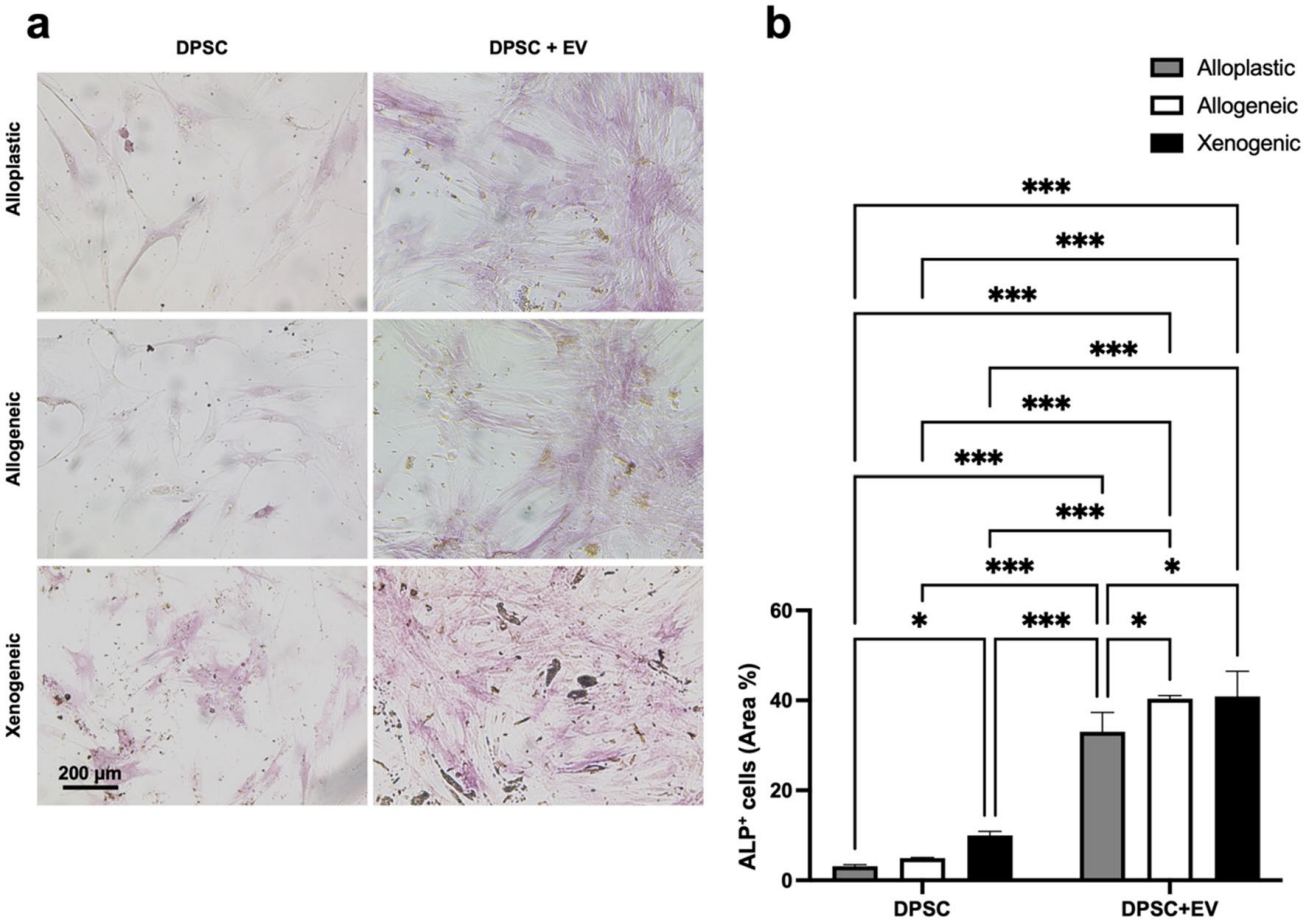
(**a**) Images of ALP staining after 7 days. 100 × Magnification. (**b**) Graphical representation of APL evaluation after 7 days. ***(*p* < 0.001); *(*p* < 0.05).

**Figure 6:**
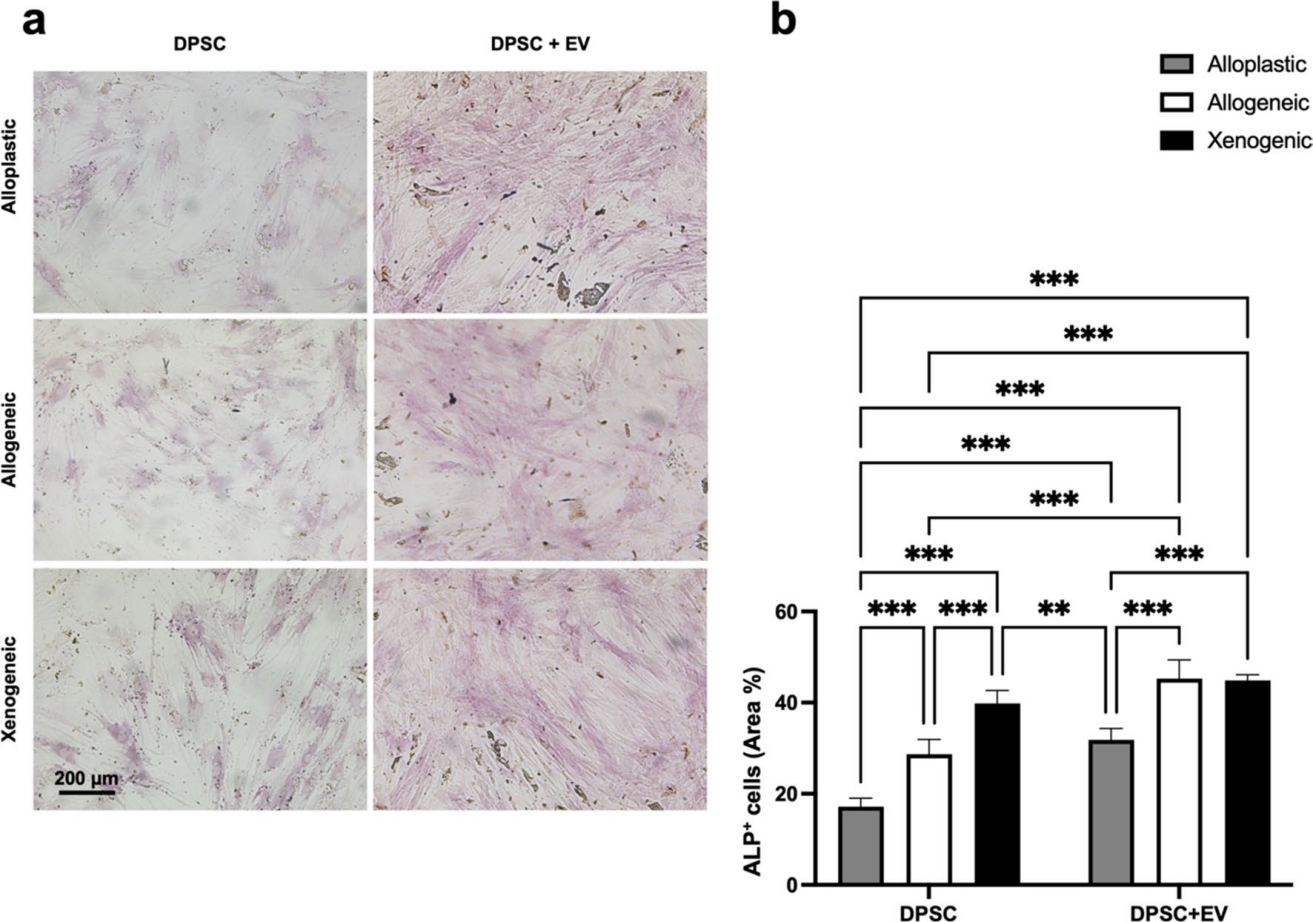
(**a**) Images of ALP staining after 14 days. 100 × Magnification. (**b**) Graphical representation of APL evaluation after 14 days. ***(*p* < 0.001); *(*p* < 0.05).

**Figure 7:**
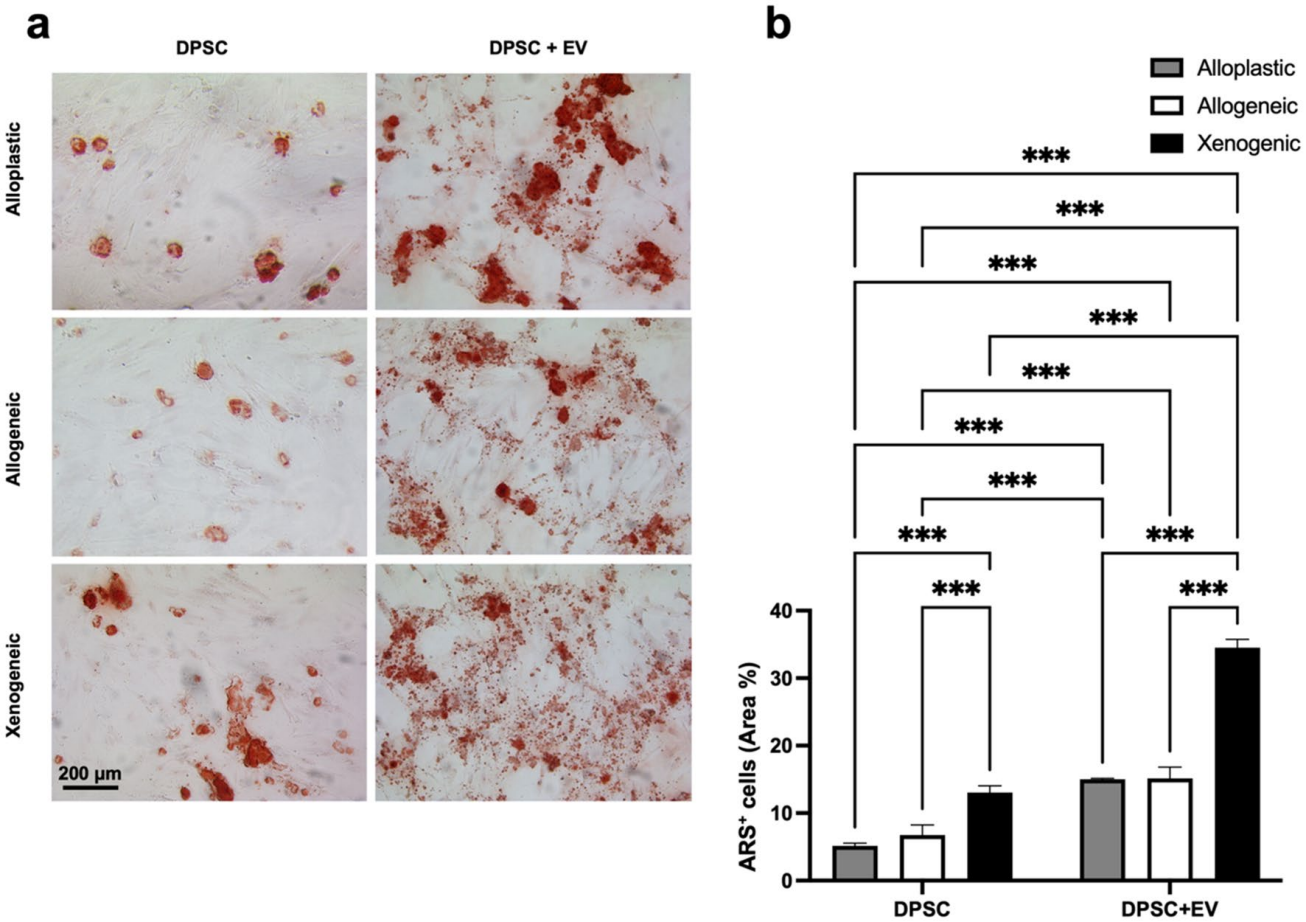
(**a**) Images of ARS staining after 14 days. 100 × Magnification. (**b**) Graphical representation of ARS evaluation after 14 days. ***(*p* < 0.001); *(*p* < 0.05).

In DPSCs, ALP activity was determined after 7- and 14-day treatment with EV and without EV stimulation as an early indicator of cell differentiation toward the osteogenic lineage. Compared to DPSC without EVs, EVs significantly (*p* < 0.001) increased ALP activity in all BGSs after 7 days of culture (Table 2 and Fig. 5a,b). The highest proportion of the 40.85 ± 5.63% of ALP+ area was found in DPSC-EVs cultured on xenogeneic BGS. After 14 days, all EV groups showed a significant increase in ALP activity compared to DPSCs without EVs. Again, DPSCs on the xenogeneic scaffold showed the highest ALP activity in the group without EVs, with a 39.81 ± 2.88% ALP+ area (*p* < 0.001), and also in the group of DPSCs + EVs, with a 44.85 ± 1.25% ALP+ area, which was significantly higher (*p* < 0.001) than the alloplastic graft DPSC + EVs, with a 31.83 ± 2.50% ALP+ area (Table 2 and Fig. 6a,b). Alizarin red staining (ARS) and Von Kossa staining were performed to evaluate the late phase of osteogenic differentiation after 14 days. Analogously to the ALP staining, a significantly increased ARS+ area was also found under the influence of EVs in all BGS materials. This effect was particularly evident in xenogeneic BGS material. Without EVs, the ARS+ area was 13.04 ± 1.02%, and with EVs the percentage significantly increased (*p* < 0.001) to an ARS+ area of 34.53 ± 1.22% (Table 2 and Fig. 7a,b).

**Table 2:** Quantitative data of osteogenic differentiation.

Groups	ALP+ area (%) 7 days	ALP+ area (%) 14 days	ARS+ area (%) 14 days
DPSC			
Alloplastic	3.13 ± 0.33	17.19 ± 1.84	5.15 ± 0.39
Allogeneic	4.91 ± 0.17	28.69 ± 3.24	6.74 ± 1.52
Xenogeneic	9.97 ± 0.92	39.81 ± 2.88	13.04 ± 1.02
DPSC + EVs			
Alloplastic	33.02 ± 4.32	31.83 ± 2.5	15.01 ± 0.15
Allogeneic	40.38 ± 0.7	45.25 ± 4.14	15.14 ± 1.69
Xenogeneic	40.85 ± 5.63	44.85 ± 1.25	34.53 ± 1.22

## Discussion

The therapeutic potential of MSC-derived EVs has already been extensively reported^9,12,15,23,25,28,31^. Both in vivo and in vitro studies have sufficiently demonstrated that many key factors of bone remodeling are controlled under the regulatory action of MSC-derived EVs^9,19,23,25^.

In this study, we focused on MSCs isolated from dental pulp tissue. Human DPSC EVs were extracted, and we compared the interaction as well as osteogenic differentiation of human DPSCs under the influence of EVs in different bone scaffolds to better understand the role of EVs derived from DPSCs on the one hand and the influence of different bone substitutes on the therapeutic effects of DPSCs on the other. All scaffolds were able to carry DPSC, and all DPSCs we tested were able to incorporate the added EVs in vitro. During TEM examination, the DPSCs in alloplastic, allogeneic, and xenogeneic scaffolds with and without EVs showed a normal shape, and no morphological differences were observed. Likewise, the monocultured DPSCs with EVs showed a homogeneous distribution of actin fibers and filament extension on all bone substitute materials used in the study.

The general ability of DPSCs to internalize EVs was an important aspect of this study, suggesting that the uptake of EVs is related to our observed effects of DPSCs. Many studies have already adequately explored miRNAs^25^, surface proteins, and glycoproteins, as well as the cell-specific pathways of EV internalization^32^. It was beyond the scope of this study to determine the efficiency of internalization of our EVs or to identify and experimentally confirm the individual miRNAs, surface proteins, and glycoproteins of the cell-specific uptake pathways on the EV surface. Our study focused primarily on the qualitative uptake of the extracted EVs into the DPSCs. Qualitative investigations of whether EVs can be internalized in target cells have already been performed by other researchers^25,32^. Over the duration of 8 h, we observed a steady internalization and accumulation of fluorescently WGA-labeled EVs into the DPSCs with DAPI-stained nuclei via CLSM in our experiments. Considering the results of the proliferation and differentiation experiments, we can assume that the EVs were internalized efficiently enough into the DPSCs.

According to Mondal et al.^32^, the isolation of EVs is a significant step for research on these vesicles. Although previous research has described disadvantages such as the increased time and potential protein contamination required for the purification of EVs by ultracentrifugation^32^, we did not observe strong contamination by non-vesicular proteins by our nano-tracking studies or by SEM examination. The heterogeneity of EV sizes is a natural phenomenon based on various intercellular signaling mechanisms of cells^33^. Previous studies have described the heterogeneous sizes of EVs between 30–200 nm^9,25,32^. In line with this earlier work, EVs in the size range of about 184.9 ± 67.0 nm were determined in our study by nano-tracking analysis. The morphological evaluation of EVs by SEM examination also showed that the isolated EVs were identical in their morphological shape, despite their heterogeneous sizes.

The treatment of craniofacial bone defects^34^ and an insufficient bone supply of the jaw pose great challenges to therapists^3,35^. Although pure bone substitutes are superior to autologous bone in terms of harvest morbidity^36^, they have significant deficiencies in terms of osteogenesis, osteoinduction, and osteoconduction^3^. A rigorous search for alternative methods is thus underway to improve the osteogenic properties of BGSs^3,7,35,37^. Re et al.^13^ described improved bone regeneration of bone substitutes when MSCs and derived EVs were added to the scaffolds. Our investigations of DPSCs were performed on alloplastic, allogeneic, and xenogeneic bone grafts, and according to Re et al.’s findings^13^, all scaffolds examined in the present study were capable of bearing DPSCs, and the influence of EVs resulted in an increased induction of osteoblastic differentiation. Nevertheless, significant differences in the amount of cell proliferation, cell attachment, and osteogenic differentiation were observed under the influence of EVs as well as in the dependence of the BGSs.

Calcein AM staining, which indicates intracellular esterase activity, is an established method for assessing the viability of DPSCs^31,35^. In our experimentation, we showed that DPSCs under the influence of EVs grown on xenogeneic scaffolds for 3 and 7 days, respectively, exhibited significantly higher numbers of attached and proliferated DPSCs, with up to 773.25 ± 5.95 CAM+ cells/mm^2^ (*p* < 0.001) compared to all other groups. This observation is consistent with the work of Zhang et al.^31^, who described in an in vivo study an increased number of calcein-positive MSCs in the presence of EVs used with xenogeneic bone material with an EV concentration of 1 × 10^12^ nanoparticles/ml versus significantly decreased calcein-positive bone in the absence of EVs. We used a lower amount of EVs in our study (2 × 10^9^ nanoparticles/ml), but despite this lower concentration of EVs, we observed a highly significant (*p* < 0.001) effect on the cell viability of DPSCs under the stimulation of EVs. While in the live/death assay the number of live DPSCs with EVs on the xenogeneic scaffold (959 ± 11.81 live cells/mm^2^) was significantly higher (*p* < 0.001) compared to all other groups, no difference was noted with a 1.88% of dead cells compared to the percentage of dead cells with the alloplastic scaffold (1.74%) and the allogeneic scaffolds (2.28%), respectively. Considered together, these findings indicate that EVs and xenogeneic BGSs favor the attachment and proliferation of DPSCs, but that no difference in biocompatibility exists between each material and the influence of EVs.

Motamedian et al.^29^ reported a significant effect of different BGSs on the in vitro cell differentiation of DPSC. By the evaluation of osteogenic differentiation by APL staining, this effect was confirmed in our studies. Compared to the alloplastic scaffold, the DPSC culture on the xenogeneic BGSs showed significantly higher ALP activity at 7 days and at 14 days, with a 9.96 ± 0.92 ALP+ area (*p* < 0.05) and a 39.81 ± 2.88% ALP+ area (*p* < 0.001), respectively. In contrast to our findings, a favorable effect on the differentiation of DPSCs by freeze-dried bone allograft has been described in the literature^29^, whereas the data of our study have shown that the xenogeneic bone material was superior to the alloplastic and autogenous scaffolds in terms of osteogenic differentiation of DPSCs. This phenomenon can possibly be explained by the use of different BGSs between the studies, which were obtained differently and varied in chemical composition. This process should be standardized for the future selection of an appropriate BGS in further in vivo clinical investigations. In a comparative study, significantly increased ALP activity was determined after 14 days in MSCs under the influence of EVs compared to treatment by an osteogenic medium in vitro^25^. Analogously, a 5.04% increase in ALP activity was observed in the DPSC group on the xenogeneic BGS under the influence of EVs, showing the strongest osteogenic differentiation among all scaffold groups after an observation period of 14 days.

In the context of bone defects and osteoporotic diseases, because researchers have focused primarily on the utilization of EVs from MSCs from bone marrow and their biologically active content^31,38^, the field lacks knowledge of the use of DPSCs as alternative cell sources to obtain EVs that have the osteogenic potential to enhance bone mineral formation. But EVs also have the potential to enhance stem cell differentiation, since they can accelerate bone mineral deposition, among other applications^9,31,39^. Zhang et al.^31^ have described a higher percentage of ARS+ cells in xenogeneic bone in the presence of EVs after 8 weeks of bone healing. Consistent with their findings, our data have demonstrated a significant increase in ARS+ cells by the presence of EVs in all groups after 21 days. Compared with all scaffolds with and without EV intervention, the DPSC-EVs grown on the xenogeneic scaffold demonstrated significantly increased osteogenic differentiation in the late phase, with an increase of 21.45% ARS+ cells.

## Conclusion

Within the limitations of an in vitro study, the data from the present work show that the effect of extracellular vesicles (EVs) on cell proliferation, cell attachment, and osteogenic differentiation of dental pulp stem cells (DPSCs) cultivated on xenogeneic scaffolding is significantly superior to that of other bone grafts (BGSs). The ease of obtaining DPSCs and the reliable isolation method of EVs, combined with the synergistic osteogenic differentiation rates, demonstrate their suitability for future bone tissue engineering studies. Further investigation of the findings of this study on the corresponding bone substitutes, as well as the osteogenic properties of DPSCs under the influence of EVs on bone regeneration, should be performed in future studies in living organisms in the form of animal experiments.

## Data Availability

All data generated for this study are available from the corresponding authors upon reasonable request.
